# Evaluation Frameworks for Predictive and Generative Oncology AI: Current Standards, Cancer-Specific Gaps, and a Path Toward Clinical Use

**DOI:** 10.3390/cancers18121981

**Published:** 2026-06-18

**Authors:** Connor D. Yost, Bradley Callas, Peter Halligan, Peter Palumbo, Samarth Rawal, Yan Leyfman, Ryan H. Nguyen

**Affiliations:** 1 Department of Internal Medicine, Creighton University School of Medicine, Phoenix, AZ 85013, USA; 2 Creighton University School of Medicine, Phoenix Regional Campus, Phoenix, AZ 85013, USA; bradleycallas@creighton.edu (B.C.); peterhalligan@creighton.edu (P.H.); 3 Geisel School of Medicine at Dartmouth, Hanover, NH 03755, USA; peter.a.palumbo.tu27@dartmouth.edu; 4 Department of Internal Medicine, Mayo Clinic, Phoenix, AZ 85054, USA; rawal.samarth@mayo.edu; 5 Division of Hematology and Medical Oncology, Meyer Cancer Center, NewYork-Presbyterian Hospital, Brooklyn, NY 11215, USA; lse9006@nyp.org; 6 Division of Hematology and Oncology, Department of Medicine, University of Illinois Chicago, Chicago, IL 60612, USA

**Keywords:** large language models, clinical decision support, oncology, response prediction, machine learning, evaluation framework, TRIPOD-LLM, ESMO ELCAP, external validation

## Abstract

Artificial intelligence (AI) is now applied across oncology, including imaging, pathology, therapy selection, toxicity assessment, and survival modeling, and large language models (LLMs) have been adopted particularly quickly. Reported physician use of AI roughly doubled between 2023 and 2026. The frameworks used to evaluate these tools have not advanced at the same rate. Many are available, but each was designed for a single stage of the AI lifecycle, and none directly answers the question most relevant to a treating clinician: whether a given tool is appropriate for the individual patient under care. We reviewed the frameworks in current use, including TRIPOD+AI, PROBAST+AI, CLAIM, SPIRIT-AI, CONSORT-AI, DECIDE-AI, MINIMAR, and CREMLS, together with the oncology-specific ESMO EBAI and the newer LLM-specific guidance (TRIPOD-LLM, MI-CLEAR-LLM, CHART, and ESMO ELCAP). Each addresses part of the evaluation problem, but none is sufficient on its own for oncology, where the standard of care changes rapidly, assays drift, biomarker-defined subgroups are small, and a model validated in one period may perform poorly in the next. LLMs introduce additional challenges, including sensitivity to prompting, undisclosed vendor updates, behavioral drift, and hallucination rates approaching 50% on clinical quality-assurance tasks. The predictive-model frameworks were not designed to capture these failure modes. The central argument of this review is that the frameworks the field requires already exist; what is missing is their mandatory adoption. We propose that journals and regulators move from recommending these frameworks to requiring their use, and we outline a cancer-aware evaluation pathway together with the specific responsibilities of authors, reviewers, journals, and regulators in implementing it.

## Simple Summary

Artificial intelligence (AI), including chatbot-style large language models, is increasingly used to help make cancer-care decisions. Many checklists and guidelines exist to judge whether such tools are trustworthy, but they are scattered and inconsistently applied, and none alone tells a doctor whether a tool is safe for a specific patient. This review explains what each existing framework does, where important gaps remain for cancer, and how the pieces fit together across a tool’s life. We argue that journals and regulators should require, not merely recommend, these checks, and we outline a practical, cancer-aware pathway for doing so.

## 1. Introduction

Artificial intelligence (AI) refers to computational systems that perform tasks ordinarily requiring human judgment, and machine learning (ML) is the subset in which models learn patterns directly from data rather than following explicitly programmed rules. In medicine these methods now span an extraordinary range of applications, from diagnostic classification and risk prediction in conditions such as polycystic ovary syndrome to noise-robust tumor delineation on neuro-oncologic imaging [1,2]. AI and ML are now embedded across oncology, including image interpretation, pathology, therapy selection, toxicity assessment, and biomarker discovery [3,4,5,6,7,8,9]. With multi-omics, digital pathology, radiomics, and longitudinal electronic health record (EHR) data, oncology has become one of the most data-rich fields in medicine [3,4,7,8], and the potential clinical benefits, from more individualized treatment to faster trial matching and earlier recognition of toxicity, are substantial [5,6,8].

The supporting evidence, however, is uneven. Most models are developed retrospectively at a single center, evaluated primarily on discrimination, and reported with limited detail on calibration, missing data, subgroup performance, transportability across sites, or behavior after deployment [10,11,12]. These omissions are consequential in oncology, where the underlying data are unstable: platforms change, treatment standards shift, outcomes accrue over time, and the most clinically important subgroups are often small and biologically distinctive [9,10,11,12].

Several frameworks have emerged to address parts of this problem. Some govern reporting, others assess risk of bias, and still others address early clinical evaluation, deployment, or regulatory clearance [13,14,15,16,17,18,19,20]. The result is a fragmented landscape in which a study may satisfy one framework while failing another, leaving reviewers to reconcile them.

Large language models (LLMs) have intensified this problem. Their use in clinical practice has risen sharply, driven in large part by chatbots and clinical assistants [21], although reported adoption figures aggregate many forms of AI assistance rather than oncology decision support specifically. Unlike earlier models, LLMs are generative, sensitive to prompt design and decoding settings, and subject to vendor updates that occur without notice, none of which the predictive-model frameworks were designed to accommodate [22,23].

This review examines the frameworks currently applied to predictive oncology AI, evaluates how well they meet cancer-specific demands, and gives particular attention to LLMs used in clinical decision support. The objective is not to replace these frameworks but to clarify what each contributes, where the gaps lie, and what a cancer-aware evaluation pathway should comprise.

## 2. Approach and Scope of This Review

This is a focused narrative review. We chose a narrative rather than systematic approach because the relevant guidance documents differ widely in scope, format, and purpose and are not suited to the predefined search and screening of a systematic review. We selected framework papers that are widely used or recently updated and bear directly on predictive or decision-support AI in oncology. These frameworks fall into six groups: reporting (TRIPOD+AI, CLAIM, MINIMAR, CREMLS); risk of bias and applicability (PROBAST+AI); clinical trials (SPIRIT-AI, CONSORT-AI, DECIDE-AI); cancer-specific guidance (the European Society for Medical Oncology [ESMO] basic requirements for AI-based biomarkers, ESMO EBAI); regulatory and lifecycle guidance (U.S. Food and Drug Administration [FDA] clinical decision support, good machine learning practice [GMLP], and FUTURE-AI); and LLM-specific guidance (TRIPOD-LLM, MI-CLEAR-LLM, CHART, ESMO ELCAP) [13,14,15,16,17,18,19,20,24,25,26,27,28,29,30,31,32]. Table 1, included at the end of this manuscript, summarizes the frameworks discussed in this review.

**Figure 1:**
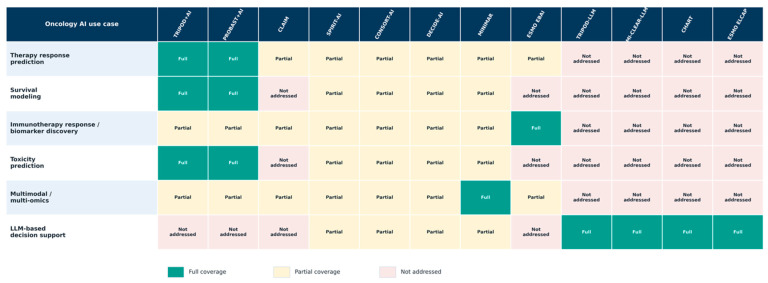
Framework coverage gap matrix across oncology AI use cases. A heatmap matrix showing how existing methodological and reporting frameworks address each oncology AI use case. Rows are common applications (therapy response prediction, survival modeling, immunotherapy response and biomarker discovery, toxicity prediction, multimodal and multi-omics modeling, and LLM-based clinical decision support). Columns are established frameworks (TRIPOD+AI, PROBAST+AI, CLAIM, SPIRIT-AI, CONSORT-AI, DECIDE-AI, MINIMAR, ESMO EBAI, TRIPOD-LLM, MI-CLEAR-LLM, CHART, ESMO ELCAP). Each cell is categorized as full coverage, partial coverage, or not addressed. The matrix shows substantial heterogeneity in framework applicability and supports the central argument that a composite, context-aware evaluation strategy is required. Coverage categories were assigned by author consensus based on each framework’s published content rather than a quantitative scoring instrument, and should be read as a structured expert assessment intended to orient readers and reviewers: full coverage indicates that the framework explicitly and adequately addresses the use case, partial coverage that it addresses it only incompletely, and not addressed that the framework does not engage the relevant requirement.

Frameworks were identified through structured but non-systematic searches of PubMed and Google Scholar, the reference lists of recent AI-evaluation reviews, and the methodological collections of reporting-guideline repositories such as the EQUATOR Network, supplemented by guidance issued by oncology and regulatory bodies. We included a framework when it was peer-reviewed or issued by a recognized professional or regulatory organization, when it addressed reporting, risk-of-bias appraisal, clinical evaluation, regulatory or lifecycle oversight, or LLM-specific assessment, and when it bore directly on predictive or decision-support AI relevant to oncology. We excluded frameworks confined to non-clinical domains or to narrow technical subtasks without an evaluation component. Frameworks aimed primarily at diagnostic test-accuracy studies, most notably QUADAS-AI, were noted where relevant to bias appraisal of diagnostic-accuracy claims but were not a central focus, because this review centers on prediction and decision support rather than diagnostic-accuracy designs [33]. The coverage judgments summarized in Table 2 and visualized in Figure 1 (full coverage, partial coverage, or not addressed) were not derived from a quantitative scoring instrument; they reflect author consensus, reached by independent review of each framework’s published content followed by discussion to resolve disagreements, and should be read as a structured expert assessment rather than a validated measurement.

These frameworks differ less in quality than in the question each was built to answer, and recognizing those differences is essential to applying them correctly. It helps to keep their distinct purposes separate: reporting standards govern completeness of description; risk-of-bias and applicability tools appraise methodological soundness; clinical-trial and early-evaluation frameworks address prospective and workflow performance; oncology-specific guidance sets evidentiary requirements for biomarkers; regulatory and lifecycle guidance covers clearance and post-market oversight; and LLM-specific guidance addresses the failure modes of generative systems. These are not interchangeable, because complete reporting does not by itself establish low risk of bias, low risk of bias does not establish clinical usefulness, and clinical usefulness in an early study does not establish readiness for sustained deployment. As shown in Table 1, TRIPOD+AI governs the completeness of reporting, specifying how predictors, outcomes, sample size, missing data, and performance should be described, but it does not establish whether a study is at risk of bias. That role belongs to PROBAST+AI, which appraises participants, predictors, outcomes, and analysis for systematic error and for applicability to the intended setting. DECIDE-AI is different again, applying only once a tool is used prospectively in a clinical workflow, where it evaluates human factors, safety, and early performance rather than reporting. SPIRIT-AI and CONSORT-AI extend trial standards to AI interventions at the protocol and reporting stages, whereas ESMO EBAI introduces oncology-specific evidentiary requirements for AI biomarkers. The LLM-specific guidance addresses problems the predictive-model tools do not, including prompt dependence, stochastic output, and model versioning [28,29,30,31,32]. A single study may therefore require several frameworks at once, each examining a different dimension of the same tool.

### Why Oncology Requires Cancer-Aware Evaluation

The frameworks above were largely designed for medical AI in general, yet several features of oncology make cancer-aware evaluation necessary rather than optional. The standard of care changes faster than in most fields, so a model trained in one treatment era can become miscalibrated as new agents redefine baseline outcomes. Molecular assays, sequencing pipelines, and imaging platforms drift, which means the inputs a model was developed on may no longer match those used in clinic. Clinically decisive subgroups, such as patients defined by a single biomarker, are often small and biologically distinctive, so pooled performance can mask failure exactly where it matters most. Endpoints are heterogeneous and time-dependent, mixing response, progression, and survival measured over long and censored follow-up. Finally, the distinction between predictive and prognostic signals, which determines whether a tool should guide a specific treatment choice, is frequently blurred in development studies. General reporting and bias tools do not interrogate these properties directly, which is why oncology AI requires evaluation that is explicitly cancer-aware.

## 3. How Current Frameworks Apply to Major Oncology AI Use Cases

### 3.1. Therapy Response Prediction and Targeted Therapy Selection

In metastatic disease, selecting the right targeted therapy at the right time is among the highest-impact decisions an oncologist makes, and a model that identified likely responders or impending resistance could shorten the time to effective treatment and reduce avoidable toxicity. Yet such models are easy to overstate: response endpoints differ between studies, regimens evolve quickly, and outcome labels can reflect clinician behavior or imaging schedules as much as tumor biology.

Hua and colleagues developed a multimodal model to predict targeted-therapy resistance in non-small cell lung cancer (NSCLC) using TRIPOD guidance [34]. The model combined imaging, pathology, and clinical features from 42 patients with epidermal growth factor receptor (EGFR) mutations receiving osimertinib, and internal cross-validation was encouraging. Limitations of the study include a small, single-center cohort and the absence of testing in patients on other regimens or at later points in the resistance course. Whether the signal would persist in community oncology practice remains uncertain.

TRIPOD+AI is a reporting standard rather than a model: it specifies how a study should describe predictor definitions, outcome timing, missing data, potential leakage, and validation design, and applied here it would make the cohort’s limitations transparent, but it cannot establish whether the model is clinically valid [13]. PROBAST+AI complements it by appraising risk of bias [14], yet neither resolves the oncology-specific questions that determine clinical utility: whether the model holds across treatment eras, whether the development assay matches the one used in clinic, and whether it predicts benefit from a specific drug or only general prognosis. When the model functions as an AI biomarker, ESMO EBAI is a stronger instrument, because it aligns the evidence required with the claim being made [24].

### 3.2. Survival Modeling

Survival prediction underlies nearly every oncology decision, from the intensity of initial treatment to the timing of palliative care discussions, and a model giving an individualized risk estimate at a defined time horizon could inform those conversations. Survival models also carry distinctive statistical demands: time origin, censoring, competing risks, treatment switching, and changing background therapy during follow-up all affect performance, none of which is captured by the area under the receiver operating characteristic curve (AUROC) or a single concordance index when the relevant questions concern horizon-specific risk, calibration, and clinical usefulness [12,35].

Causio and colleagues developed a survival model for patients undergoing radical cystectomy for bladder cancer, following the TRIPOD+AI checklist [36]. Discrimination was strong, but the authors reported a mean absolute error in time-to-event prediction high enough that they judged the tool, in their words, “unsuitable for precise individual patient counseling or treatment planning where accurate timing is critical.” In other words, the model ranked patients by relative risk effectively, yet the predicted survival times were not accurate enough to quote to an individual patient. This dissociation between strong discrimination and poor calibration recurs throughout oncology survival modeling.

TRIPOD+AI strengthens outcome definitions and validation reporting, and PROBAST+AI identifies risk of bias, but neither specifies how a survival model should be calibrated across the time horizons relevant to cancer decisions, how competing risks should be handled, or what external validation across treatment eras should entail.

### 3.3. Immunotherapy Response and Biomarker Discovery

Checkpoint inhibitors have improved survival in many solid tumors, yet most patients do not respond, and established biomarkers such as programmed death-ligand 1 (PD-L1) and tumor mutational burden (TMB) remain imprecise. AI tools incorporating richer phenotypic data could identify responders earlier, but the principal difficulty is generalization: a model developed on one dataset frequently fails on another. Such models almost always overlap with existing biomarkers, and the ground truth itself shifts with disease site, regimen, and timing of assessment.

Lee and colleagues validated a machine learning model for immunotherapy response in head and neck squamous cell carcinoma using the original TRIPOD framework [37]. The model performed well within head and neck cancer, but the authors were explicit about its limits, noting “decreased performance in a specific cancer type” and acknowledging that they “failed to assess the predictor geographical or temporal validity.” This is the decisive gap: a model that performs well in one tumor type at a single institution has not yet demonstrated the cross-site, cross-time validity that its intended clinical use would require.

ESMO EBAI is particularly well suited to this setting because it classifies AI biomarkers by function, distinguishing the measurement of a known biomarker, the indirect screening for one, and the proposal of a new predictive or prognostic biomarker, each of which demands a different evidence package [24]. Reporting checklists alone cannot determine whether a signal represents a genuine predictive biomarker, a weak surrogate, or a chance association.

### 3.4. Toxicity Prediction

Severe toxicities drive hospitalizations, dose reductions, and treatment discontinuation, and they remain difficult to predict. A model identifying high-risk patients before treatment could inform supportive care, monitoring intensity, and the framing of informed consent. Such models are highly sensitive to how the endpoint is defined, so the same patient may be labeled differently across sites depending on whether the outcome is any grade of toxicity, grade 3 or higher, or treatment discontinuation.

TRIPOD+AI, PROBAST+AI, and MINIMAR help define outcomes and structure validation [13,14,19], but they do not require the workflow analysis that separates a clinically useful toxicity model from one that has merely learned to reproduce a chart label or that generates alert fatigue. DECIDE-AI is the framework built for this stage [18]. Rather than assessing reporting or risk of bias, it evaluates a tool during early, small-scale clinical use, addressing human factors, the safety of human-AI interaction, the balance of actionable against spurious alerts, and the consequences of acting on the model’s output. These elements determine whether a statistically accurate prediction actually improves care, and they become apparent only once the tool is embedded in a real workflow.

### 3.5. Multimodal and Multi-Omics Systems

Cancer is biologically heterogeneous, encompassing imaging, histology, molecular features, and clinical course, data that oncologists already synthesize at tumor board. Multimodal AI attempts this integration more systematically and can reveal signals no single data type would expose, but it often fails unpredictably. Each additional modality multiplies the potential for missing data, batch effects, harmonization difficulties, and confounding, so a model that performs well on a complete dataset may deteriorate when one modality is absent, measured differently, or acquired on different hardware.

No current framework addresses this situation adequately. MINIMAR and TRIPOD+AI improve reporting and PROBAST+AI flags general threats to validity, but none specifies how a multimodal oncology tool should handle missing modalities, perform across platforms, or accommodate the feature shifts introduced by different preprocessing pipelines. This remains one of the clearest areas in which cancer-specific extensions are still required.

Because multimodal and multi-omics systems are likely to become central to oncology, this gap deserves dedicated attention rather than incidental mention. A cancer-specific extension for these systems would need to specify, at minimum, how performance is reported when one or more modalities are missing at inference, how models are validated across scanners, stains, and sequencing platforms, how batch effects and harmonization steps are documented, and how the marginal contribution of each modality is demonstrated rather than assumed. Until such guidance is developed, reviewers can still require the substance of it: explicit reporting of missing-modality behavior, per-site and per-platform performance, and an ablation showing that the multimodal model outperforms its strongest single-modality counterpart. Developing consensus on these points is among the more pressing directions for future framework work in oncology AI.

### 3.6. Large Language Models for Clinical Decision Support

Many oncology decisions are documented in unstructured text, including clinic notes, multidisciplinary recommendations, changing guidelines, and trial eligibility criteria. LLMs that summarize a chart, answer a question during a visit, or surface treatment options at the point of care have already moved from demonstration to pilot use, which raises the central question of how such tools should be validated before they influence care.

LLMs used for clinical decision support are neural-network systems trained on large text corpora that interact with clinicians in real time to support note review, treatment planning, and trial matching. The American Medical Association reported that the share of physicians using AI in clinical practice rose from 38% in 2023 to 81% in 2026 [21], although that figure spans a broad range of applications, including documentation and scheduling tools, rather than diagnostic or decision-support AI alone. The interest is real, and so is the hesitation: clinicians and informaticians worry about thin validation, hallucinations, and ethical and legal exposure [38].

The older prediction frameworks do not fit this kind of tool. TRIPOD+AI and PROBAST+AI assume a fixed dataset with defined inputs and outputs, whereas clinical decision support LLMs are generative, evolve quickly, and produce outputs that depend on prompt design, surrounding context, and decoding parameters such as temperature [22,23]. The newer LLM-specific guidelines, TRIPOD-LLM, CHART, and MI-CLEAR-LLM, emerged to fill that gap [28,29,30,31]. For oncology specifically, ESMO ELCAP groups applications as patient-facing (Type 1), clinician-facing (Type 2), and background institutional (Type 3), each with its own validation requirements [32]. Most decision support tools fall into Type 2, where the LLM directly shapes clinician decisions and the validation bar should be highest.

The newer frameworks share the weaknesses of the older ones. Reporting quality has not improved since TRIPOD-LLM was published, adherence to MI-CLEAR-LLM is as poor as adherence to earlier guidelines, and whether ESMO ELCAP changes what gets reported in oncology LLM papers has not been tested [39,40]. In one analysis of ontology-grounded clinical question-answering tasks, baseline hallucination rates reached 48 to 63 percent [41]. For decision support, where a single hallucinated dose or eligibility criterion can flip a treatment decision, that rate is far too high for unsupervised use; mitigation techniques exist, but no guideline requires hallucination assessment. Several mitigation strategies are nonetheless maturing, including retrieval-augmented generation, ontology and knowledge-graph grounding, self-consistency and ensemble decoding, constrained or structured output, and human-in-the-loop verification, and newer model generations with stronger reasoning and tool use have lowered error rates on some clinical benchmarks. These advances narrow the gap but do not close it, and none removes the need for locally validated reference standards and recurring audits. Decoding parameters and versioning can produce large output swings for the same prompt, yet one analysis of radiology LLM studies found that only 17.2% reported the settings governing stochasticity, only 43.0% provided full prompts, and only 21.5% specified the model version and access date [39]. Commercial models are also updated silently by vendors, which can invalidate a published result within weeks [39].

The deeper problem is pace. In one study, a widely used commercial model fell from 84% to 51% accuracy on a simple task over a four-month window, which the authors attributed to behavior drift and the lack of transparency around vendor updates; the same model, they noted, can “change substantially in a relatively short amount of time” [42]. For clinical decision support, this means the tool validated this quarter is not guaranteed to behave the same way the next.

The clinical takeaway is hard to escape: validating an LLM-based decision support tool once is not enough. Oncology programs deploying these tools for triage, summarization, biomarker interpretation, trial matching, or guideline lookup need locally curated reference standards, version-pinned reporting, structured prompt logs, and recurring hallucination audits before, during, and after rollout.

## 4. Persistent Gaps in the Current Framework Landscape

When these frameworks are applied to actual oncology AI, several issues recur consistently. Table 2 summarizes how each oncology use case maps to currently available frameworks and where the open gaps sit, and Figure 1 displays the same coverage as a heatmap so that the partial-coverage cells are visually identifiable at a glance.

Reporting compliance is not the same as methodologic soundness. A paper can satisfy every TRIPOD+AI item and still rest on weak outcome labels, a mismatched validation cohort, or undetected data leakage, just as strong methods can be obscured by weak reporting on what would matter for deployment.

Evaluation is fragmented. No single tool tells a reviewer at once whether an oncology AI system is well reported, low in bias, clinically relevant, fairly tested across subgroups, prospectively studied, and ready for routine use. This is part of why the field remains stuck in the so-called AI chasm between strong development papers and tools that are actually used [35,43].

Prospective evidence is thin. A recent systematic review of AI in post-diagnosis cancer pathways found that most of the literature is still experimental, with sparse prospective validation and limited analysis of rollout [35]. That gap matters most for exactly the tools the field most wants, those that change treatment selection or biomarker interpretation.

Post-rollout is the weakest stage. Oncology AI tools drift as scanner hardware changes, pathology stains evolve, sequencing pipelines update, practice shifts, and new therapies redefine the baseline, none of which a one-time development paper captures. For LLMs in clinical decision support the problem is sharper still, because a vendor can change the underlying model without anyone coordinating a revalidation [42].

Equity and subgroup reliability are uneven. Rare molecular subgroups, underrepresented racial and ethnic populations, and lower-resource sites are precisely the groups most likely to be excluded from development data or pooled into unstable estimates. Even when frameworks ask for subgroup reporting, they rarely define what counts as adequate.

These concerns are amplified in low- and middle-income countries. Frameworks such as TRIPOD+AI and PROBAST+AI implicitly assume access to large, well-curated datasets and to multiple centers with comparable equipment, conditions that frequently do not hold in global oncology, where external validation is constrained by limited multi-institutional infrastructure, heterogeneous scanners and assays, and fragmented electronic records. Applying these frameworks faithfully in such settings may require adapted expectations, including acceptance of well-documented single-center or geographically staged external validation, federated or privacy-preserving evaluation across institutions, explicit reporting of the local data environment, and local recalibration before use. Without such adaptation, rigid framework adherence risks excluding precisely the settings where validated oncology AI could add the most value, and any move from recommendation to requirement should make room for these realities.

LLM-specific failure modes are still not integrated into the general frameworks. Hallucination assessment, prompt sensitivity, decoding-parameter disclosure, version pinning, and behavior-drift checks are covered only patchily across TRIPOD-LLM, MI-CLEAR-LLM, CHART, and ESMO ELCAP, and are almost entirely absent from the prediction-model frameworks that drive most oncology peer review [28,29,30,31,32,39,40,41].

## 5. From Recommendations to Requirements: A Journal and Regulator Mandate

The evaluation problem in oncology AI is largely one of coordination rather than discovery. The frameworks already exist, but few parties are required to apply them. Journals and regulators have recommended them for several years without a corresponding improvement in reporting quality. The most consequential change available to the field over the next two to three years is to move from recommending these frameworks to requiring their use. The contribution of this review is therefore not another framework but an explicit, claim-indexed way of composing the frameworks that already exist: it specifies which instruments apply to a given clinical claim and lifecycle stage and assigns responsibility for enforcing them, which is the value the individual frameworks, taken separately, do not provide.

**Figure 2:**
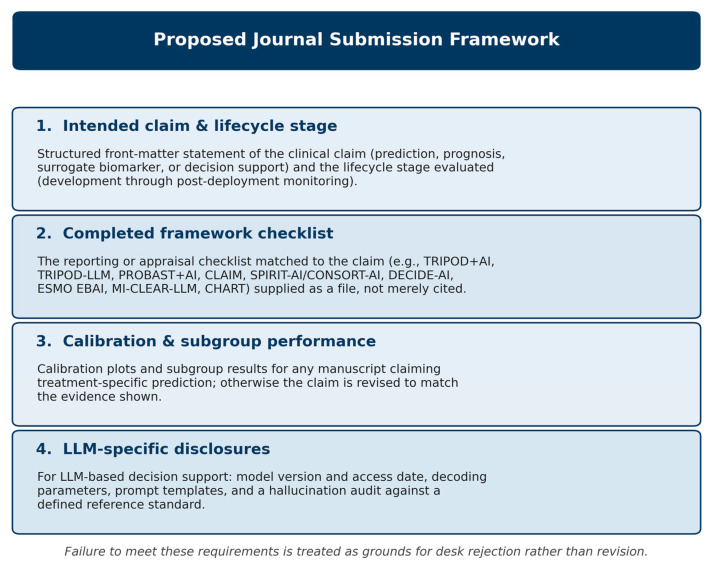
Proposed journal submission requirements for predictive and decision-support oncology AI. Four requirements that link the evidence presented in a manuscript to the clinical claim being made. (1) A structured front-matter statement declaring the intended clinical claim (prediction, prognosis, surrogate biomarker, or clinical decision support) and the AI lifecycle stage evaluated, from development through post-deployment monitoring. (2) The completed reporting or appraisal checklist appropriate to the claim (for example, TRIPOD+AI, TRIPOD-LLM, PROBAST+AI, CLAIM, SPIRIT-AI, CONSORT-AI, DECIDE-AI, ESMO EBAI, MI-CLEAR-LLM, or CHART), supplied as a file rather than cited. (3) Calibration plots and subgroup performance for any manuscript claiming treatment-specific prediction. (4) For LLM-based decision support, the model version and access date, decoding parameters, prompt templates, and a hallucination audit against a defined reference standard. Failure to meet these requirements is treated as grounds for desk rejection rather than revision. In brief, the figure asks each submission to state what the tool claims to do, attach the reporting or appraisal checklist that matches that claim, show how well the predictions are calibrated within important patient subgroups, and, for generative tools, document the exact model and prompts used together with a test of how often the tool produces incorrect statements.

For clinical journals, we propose four submission requirements that link the evidence presented to the claim being made, summarized in Figure 2: a structured statement of the intended clinical claim and lifecycle stage; the completed framework checklist for that claim, supplied as a file rather than cited; calibration and subgroup performance for any treatment-specific prediction; and, for LLM-based decision support, the model version, decoding parameters, prompt templates, and a hallucination audit against a defined reference standard. Figure 2 makes the consequence of non-compliance explicit: failure to meet these requirements should be treated as grounds for desk rejection rather than revision. Voluntary adherence has not, by itself, been sufficient to ensure consistent reporting and evaluation quality.

A practical objection to mandatory checklists is that someone must verify them, and peer reviewers rarely have time to confirm every item of a long instrument such as TRIPOD+AI. One way to make the requirement workable is to shift first-pass verification away from human reviewers. Journals could deploy automated or AI-assisted screening at submission to confirm that the declared claim, the matching checklist, and the required artifacts (calibration plots, subgroup results, prompt logs, and version metadata) are present and internally consistent, flagging omissions before a manuscript reaches a human reviewer. Such triage would not replace expert judgment on methodological soundness, but it would reduce reviewer burden, standardize the minimum every submission must contain, and let reviewers concentrate on the substantive questions that automation cannot answer. Any such tool would itself need validation and human oversight, but it offers a realistic route to enforcing checklists at scale.

The same logic applies to regulators. The FDA’s clinical decision support guidance and GMLP principles move in this direction but stop short of requiring framework alignment [25,26]. Clearance pathways should be matched to the claim: a tool that quantifies an established biomarker requires different evidence than one proposing a new predictive biomarker, and a clinician-facing LLM requires more still. Submissions for decision-support clearance should map their evidence to ESMO ELCAP for LLMs or ESMO EBAI for AI biomarkers, and post-market study commitments should be a condition of clearance rather than an optional follow-up, particularly for models that can change after deployment.

**Figure 3:**
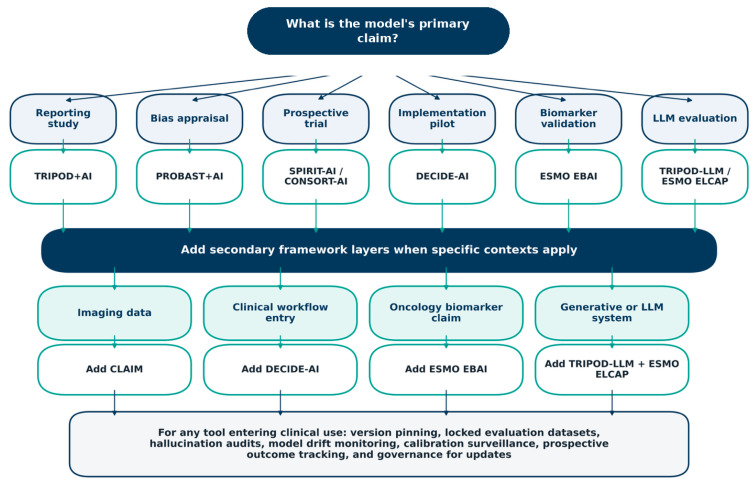
Layered evaluation pathway for oncology artificial intelligence models. A decision-based pathway for selecting evaluation frameworks based on the model’s primary claim. Entry begins with the intended use case (reporting study, bias appraisal, prospective trial, implementation pilot, biomarker validation, or LLM evaluation), each mapped to a primary framework (TRIPOD+AI, PROBAST+AI, SPIRIT-AI/CONSORT-AI, DECIDE-AI, ESMO EBAI, TRIPOD-LLM/ESMO ELCAP). Secondary layers add context-specific frameworks for imaging (CLAIM), workflow entry (DECIDE-AI), oncology biomarker claims (ESMO EBAI), and generative systems (TRIPOD-LLM, ESMO ELCAP). Terminal lifecycle requirements include version pinning, locked evaluation datasets, hallucination audits, drift monitoring, prospective outcome tracking, and update governance. Read from the top, the figure starts with what the study is mainly claiming and routes it to the framework built for that claim, then adds further frameworks only when the study also involves imaging, entry into a clinical workflow, an oncology biomarker, or a generative model. The transition between layers is governed by entry triggers, most importantly successful external validation before a tool moves from development reporting to clinical evaluation, so that a tool is not advanced toward deployment until it has met the standard for the prior stage.

Within such a mandate, the layered pathway already exists. Figure 3 shows that pathway in full. Development reporting follows TRIPOD+AI or TRIPOD-LLM, specifying predictors, preprocessing, missing-data handling, outcome definitions, calibration metrics, and, for LLMs, the relevant prompt and decoding settings [13,29]. PROBAST+AI governs bias review [14], CLAIM adds image-specific detail where relevant, DECIDE-AI covers early clinical evaluation, and SPIRIT-AI and CONSORT-AI govern trials [15,16,17,18]. ESMO EBAI applies to AI biomarkers, ESMO ELCAP to LLMs, and CHART and MI-CLEAR-LLM to chatbot and accuracy reporting [30,31,32].

The pathway shown in Figure 3 is a conditional sequence rather than a fixed checklist. The entry point is always the model’s primary claim, which determines the governing framework; additional layers are then added according to context, such as imaging data (CLAIM), entry into a clinical workflow (DECIDE-AI), an oncology biomarker claim (ESMO EBAI), or a generative system (TRIPOD-LLM and ESMO ELCAP). Not every tool requires every layer; the objective is adequate coverage of reporting, bias, clinical relevance, and lifecycle, rather than the accumulation of checklists.

To make the transitions between layers concrete, each layer has an entry trigger rather than an arbitrary handoff. A model remains in the development-and-reporting layer (TRIPOD+AI or TRIPOD-LLM, with PROBAST+AI appraisal) until it demonstrates adequate, prespecified performance on genuinely external data, meaning acceptable discrimination together with calibration in an independent cohort drawn from a different site or treatment era; meeting that threshold is the trigger to enter early clinical evaluation under DECIDE-AI, where the question shifts from statistical performance to human factors, safety, and workflow effect. A further trigger, evidence of benefit and safety in supervised clinical use, justifies prospective trial evaluation under SPIRIT-AI and CONSORT-AI. Defining these thresholds in advance keeps tools from skipping from retrospective development directly to deployment.

For authors, reviewers, and editors, the governing frameworks can be selected directly from a manuscript’s primary claim: prediction-model development calls for TRIPOD+AI and PROBAST+AI; imaging or radiomics studies add CLAIM; biomarker claims add ESMO EBAI; early clinical-workflow use adds DECIDE-AI; prospective trials add SPIRIT-AI and CONSORT-AI; and LLM-based systems add TRIPOD-LLM, MI-CLEAR-LLM, CHART, and ESMO ELCAP according to the use case. This claim-to-framework mapping is the operational core of the pathway in Figure 3 and is what allows it to be used directly in peer review and editorial practice.

Figure 1 makes the size of the remaining problem visible. It maps current framework coverage across the major oncology AI use cases and exposes where the open gaps cluster, with multimodal systems and LLM-based decision support standing out as the areas with the thinnest coverage across the existing toolkit.

Beyond manuscripts and clearance, deployed clinical tools require lifecycle evaluation, including defined intended use, attention to human factors, update governance, transparency, drift monitoring, and plans for local recalibration or retraining where appropriate [25,26,27], with scheduled hallucination audits and version control for LLM-based tools. None of this is novel; what must change is responsibility for enforcement, and journals and regulators are the field’s most direct levers.

## 6. Practical Recommendations for Authors, Reviewers, and Journals

For authors, the work begins with matching the claim to the evidence. A tool presented as a treatment-response predictor should not rest on retrospective discrimination alone if it is functionally prognostic or if treatment-specific transportability has not been tested. Calibration, subgroup performance, and a clear statement of intended use belong in every such paper, not only those a reviewer happens to query. For LLM-based decision support, the requirements are higher still, extending to the model version and access date, decoding settings, prompt templates, and the observed rate and type of hallucinations.

For reviewers, a small set of questions identifies most deficiencies: whether the study is clearly reported, whether its design is at low risk of bias, whether the endpoint matches the clinical claim, whether the model has been tested under conditions resembling real use, and whether evidence exists beyond retrospective internal validation. For LLM submissions, three additional questions apply: whether the model version and prompts can be reproduced, whether stochasticity was reported, and whether hallucinations were assessed against a defined reference standard.

For journals, Section 5 establishes the minimum standard. In routine practice, the expectations to enforce are mandatory framework selection at submission, calibration reporting, precise external-validation language, and disclosure of code or model-card availability where feasible. For LLM-based decision-support submissions specifically, editors should be empowered to request a hallucination audit even when reviewers do not.

Beyond submission practice, several steps would strengthen the field. There is still no cancer-specific consensus for multimodal and multi-omics models, and expectations for prospective silent trials and pilot deployments, particularly in biomarker-defined subgroups, remain inconsistent. The thresholds at which a model should be recalibrated, locally validated, or fully revalidated after moving to a new site or treatment era have never been clearly defined. For LLM-based decision support, the field still lacks standardized hallucination benchmarks, agreed standards for version and prompt reporting, and shared reference datasets for cross-site comparison [32,40].

## 7. Future Directions for Review and Synthesis

This review describes a rapidly changing field, and several questions could not be resolved here. An updated systematic review of oncology AI reporting quality will be warranted once TRIPOD-LLM and the other LLM-specific frameworks have been in use long enough for at least two reporting cycles to elapse; until then, whether the newer frameworks change practice remains an open question.

Comparative reviews across cancer types would also be valuable, since the framework mismatches described in Section 3 may not be uniform. Settings such as head and neck cancer, immunotherapy-predominant disease, and lung cancer with rapid molecular evolution may prove to be particularly poorly served by current evaluation tools.

Reviewer training is almost entirely unstudied. A useful next step would be to determine whether structured AI-methods training for peer reviewers alters acceptance decisions or the revisions requested, and whether journals that adopt mandatory framework selection publish higher-quality oncology AI work.

Post-deployment monitoring is the least studied stage of the lifecycle. Reviews or prospective registries that document how oncology AI tools perform after deployment, including how often they are recalibrated, retired, or replaced, would establish realistic lifecycle expectations.

Cancer-specific extensions of general AI frameworks should continue to be developed. ESMO EBAI demonstrates that cancer-specific consensus is both achievable and useful [24], and comparable guidance is likely needed for response prediction, toxicity modeling, multimodal systems, and LLM-based decision support. Future reviews should track whether these efforts converge or whether the field fragments further.

### Limitations of This Review

This review has several limitations. As a narrative rather than systematic review, framework selection reflects the authors’ judgment and may not capture every relevant guidance document. The landscape of AI evaluation frameworks is changing quickly, so assessments of real-world adherence and impact rest on the literature available at the time of writing and may date within months. The proposed layered pathway is conceptual and has not been prospectively validated against clinical outcomes or reviewer agreement, and the review focuses primarily on frameworks for the peer-reviewed literature rather than the regulatory and post-market surveillance pathways used for commercially deployed tools.

## 8. Conclusions

LLMs are increasingly used by many oncologists, most often for documentation and information tasks rather than autonomous treatment decisions, even as other forms of predictive AI are adopted unevenly across cancer care. The existing frameworks have been valuable but remain incomplete, particularly for LLM-based clinical decision support. No single framework, on its own, establishes whether an oncology AI tool is transparent, unbiased, clinically useful, and ready for routine use.

A layered, cancer-aware pathway, encompassing careful reporting, bias review, prospective evaluation, clearly defined intended use, ongoing monitoring, and LLM-specific safeguards, is the most realistic way forward, but such pathways function only when enforced. The change most likely to alter practice is a shift by journals and regulators from recommendation to requirement. For LLM-based decision support, evaluation cannot be a single benchmark; it requires version pinning, reporting of prompts and decoding settings, and hallucination audits after each vendor update. LLMs are increasingly used in tasks adjacent to response prediction, treatment selection, and patient communication in oncology, though their influence on definitive treatment decisions remains variable and frequently supervised, and the standard of evidence must rise accordingly.

In practice, the pathway reduces to a sequence any author, reviewer, or editor can follow: define the clinical claim and lifecycle stage; select the matching frameworks; report development completely; appraise risk of bias; demonstrate calibration and subgroup performance; provide prospective or workflow evidence where the claim requires it; and commit to post-deployment monitoring, with version pinning, prompt and decoding disclosure, and recurring hallucination audits for LLM-based tools. Making each of these steps a requirement rather than a recommendation is the single change most likely to raise the quality of oncology AI evidence.

## Figures and Tables

**Table 1:** Main frameworks used to judge predictive oncology AI systems.

Framework	Main Purpose	Best Fit in Oncology	Key Strengths	Important Limits
TRIPOD+AI	Clear reporting of diagnostic and prognostic prediction model studies that use regression or ML	Response prediction, survival prediction, clinical EHR-based models	Better reporting of datasets, predictors, outcomes, modeling, and validation	Not a bias tool; does not set clinical readiness or rollout criteria
PROBAST+AI	Quality, bias, and fit check for prediction models	Reviewing published predictive oncology models before clinical use	Strong focus on leakage, overfitting, outcome definitions, and validation	Does not set rollout thresholds or operational workflows
CLAIM	Reporting of AI studies in medical imaging	Radiology, radiomics, and image-based pathology workflows	Image-specific details, dataset description, error and failure analysis	Limited fit for multi-omics or non-imaging clinical prediction
SPIRIT-AI	Reporting of trial protocols that use AI tools	Prospective oncology trial protocols testing AI tools	Clearer reporting of AI tools, setting, inputs and outputs, and human-AI interaction	Only applies when a prospective trial is being designed
CONSORT-AI	Reporting of trial reports that use AI tools	Prospective oncology trials of AI-assisted care or tools	Better transparency of trial conduct and AI-related details	Does not cover pre-trial development or post-rollout monitoring
DECIDE-AI	Reporting of early clinical evaluation of AI decision-support tools	Silent trials, pilot rollouts, workflow studies, exploratory use	Helps move from research to clinic; focuses on real-world context	Not meant to replace reporting or bias tools for development studies
MINIMAR	Minimum information standard for medical AI reporting	Wide baseline checklist across many oncology AI studies	Simple cross-domain structure; covers target population and validation context	Too thin alone for complex multimodal oncology systems
CREMLS	Reporting of prognostic and diagnostic ML modeling studies	Backup reporting framework for diagnostic and prognostic oncology ML	Useful for method description and standardization	Heavy overlap with TRIPOD+AI; limited rollout guidance
ESMO EBAI	Cancer-specific validation framework for AI biomarkers	Digital pathology biomarkers, radiology biomarkers, response and prognostic biomarker models	Adds cancer-specific evidence logic and intended-use grouping	Focused on AI biomarkers rather than all oncology AI tools
FDA CDS, GMLP, lifecycle guidance	Rollout and regulatory framing for clinical AI tools	AI decision support nearing real clinical use	Clearer intended use, transparency, human factors, lifecycle monitoring	Not a manuscript reporting checklist; may not map cleanly onto academic review
TRIPOD-LLM	Reporting of studies that use large language models	Oncology LLM evaluations including triage, summary, Q&A, and decision support	Adapts TRIPOD ideas to generative tools; covers prompt and version reporting	Adoption is early; effect on reporting quality not yet shown
MI-CLEAR-LLM	Minimum reporting items for LLM accuracy studies in healthcare	Accuracy and benchmarking studies of oncology LLMs	Concrete checklist items for stochasticity, prompts, and version reporting	Use in published studies remains low
CHART	Reporting guideline for chatbot health advice studies	Patient-facing oncology chatbots and triage assistants	Tailored to chatbot use and health-advice settings	Focused on chatbots rather than wider clinical LLM use
ESMO ELCAP	ESMO guidance on LLMs in clinical practice	Grouping oncology LLM use cases and validation needs	Cancer-specific grouping (Type 1, 2, 3) and validation logic	Real-world effect on reporting quality not yet checked

**Table 2:** Cancer-specific evaluation needs that are not fully met by current general AI frameworks.

Use Case	Questions the Manuscript Should Ask	Frameworks That Help Most	Open Gap
Therapy response prediction	Is the endpoint treatment-specific? Is calibration kept across sites and treatment eras?	TRIPOD+AI, PROBAST+AI, EBAI	Prediction versus prognosis is often unclear; changing standard of care is not well covered
Survival modeling	Are time origin, censoring, and competing risks handled correctly?	TRIPOD+AI, PROBAST+AI	Limited cancer-specific guidance on horizon-specific calibration and treatment-era drift
Immunotherapy response	Is the AI output a stand-in for a known biomarker or a new predictor?	EBAI, TRIPOD+AI, PROBAST+AI	Unstable ground truth and biological interpretability are not fully standard
Toxicity prediction	Are adverse events defined the same way, and does the tool improve care in workflow?	TRIPOD+AI, PROBAST+AI, DECIDE-AI	Prospective clinical use and alert-burden effects are often missing
Multi-omics and multimodal models	How well does the model handle missing modalities, batch effects, and platform changes?	MINIMAR, TRIPOD+AI, PROBAST+AI	No agreed framework fully covers multimodal use across sites
AI-based biomarkers	What level of evidence is needed for routine use?	EBAI, FDA and GMLP lifecycle guidance	Prospective validation is resource-heavy and used unevenly
LLMs for clinical decision support	What model version, prompt template, and decoding settings were used? Were hallucinations checked against a reference standard?	TRIPOD-LLM, MI-CLEAR-LLM, CHART, ESMO ELCAP	Hallucination benchmarks, version pinning, and post-update rechecks are still early

## Data Availability

No new data were created or analyzed in this study. All frameworks, guidelines, and literature discussed are cited and publicly available through the references listed.

## References

[1] Ghaderzadeh M., Garavand A., Salehnasab C. (2025). Artificial intelligence in polycystic ovary syndrome: A systematic review of diagnostic and predictive applications. BMC Med. Inform. Decis. Mak..

[2] Porkar P., Mehrabipour F., Pourasad M.H., Movassagh A.A., Nazari K. (2025). Enhancing cancer zone diagnosis in MRI images: A novel SOM neural network approach with block processing in the presence of noise. Iran. J. Blood Cancer.

[3] Wu T., Duan Y., Zhang T., Tian W., Liu H., Deng Y. (2022). Research trends in the application of artificial intelligence in oncology: A bibliometric and network visualization study. Front. Biosci..

[4] Elemento O., Leslie C., Lundin J., Tourassi G. (2021). Artificial intelligence in cancer research, diagnosis and therapy. Nat. Rev. Cancer.

[5] Meropol N.J., Donegan J., Rich A.S. (2021). Progress in the application of machine learning algorithms to cancer research and care. JAMA Netw. Open..

[6] Kann B.H., Hosny A., Aerts H.J.W.L. (2021). Artificial intelligence for clinical oncology. Cancer Cell.

[7] McKenzie M., Irac S.E., Chen Z., Moradi A., Jenner A., Nguyen Q., Rashidieh B. (2026). Integrative spatial omics and artificial intelligence: Transforming cancer research with omics data and AI. Semin. Cancer Biol..

[8] Riaz I.B., Khan M.A., Osterman T.J. (2025). Artificial intelligence across the cancer care continuum. Cancer.

[9] El Naqa I., Karolak A., Luo Y., Folio L., Tarhini A.A., Rollison D., Parodi K. (2023). Translation of AI into oncology clinical practice. Oncogene.

[10] Corti C., Cobanaj M., Marian F., Dee E.C., Lloyd M.R., Marcu S., Dombrovschi A., Biondetti G.P., Batalini F., Celi L.A. (2022). Artificial intelligence for prediction of treatment outcomes in breast cancer: Systematic review of design, reporting standards, and bias. Cancer Treat. Rev..

[11] Finlayson S.G., Subbaswamy A., Singh K., Bowers J., Kupke A., Zittrain J., Kohane I.S., Saria S. (2021). The clinician and dataset shift in artificial intelligence. N. Engl. J. Med..

[12] Van Calster B., McLernon D.J., van Smeden M., Wynants L., Steyerberg E.W. (2019). Calibration: The Achilles heel of predictive analytics. BMC Med..

[13] Collins G.S., Moons K.G.M., Dhiman P., Riley R.D., Beam A.L., Van Calster B., Ghassemi M., Liu X., Reitsma J.B., van Smeden M. (2024). TRIPOD+AI statement: Updated guidance for reporting clinical prediction models that use regression or machine learning methods. BMJ.

[14] Moons K.G.M., Damen J.A.A., Kaul T., Hooft L., Andaur Navarro C., Dhiman P., Beam A.L., Van Calster B., Celi L.A., Denaxas S. (2025). PROBAST+AI: An updated quality, risk of bias, and applicability assessment tool for prediction models using regression or artificial intelligence methods. BMJ.

[15] Tejani A.S., Klontzas M.E., Gatti A.A., Mongan J.T., Moy L., Park S.H., Kahn C.E. (2024). CLAIM 2024 Update Panel. Checklist for Artificial Intelligence in Medical Imaging (CLAIM): 2024 update. Radiol. Artif. Intell..

[16] Cruz Rivera S., Liu X., Chan A.W., Denniston A.K., Calvert M.J. (2020). Guidelines for clinical trial protocols for interventions involving artificial intelligence: The SPIRIT-AI extension. Nat. Med..

[17] Liu X., Cruz Rivera S., Moher D., Calvert M.J., Denniston A.K. (2020). SPIRIT-AI and CONSORT-AI Working Group. Reporting guidelines for clinical trial reports for interventions involving artificial intelligence: The CONSORT-AI extension. Nat. Med..

[18] Vasey B., Nagendran M., Campbell B., Clifton D.A., Collins G.S., Denaxas S., Denniston A.K., Faes L., Geerts B., Ibrahim M. (2022). Reporting guideline for the early-stage clinical evaluation of decision support systems driven by artificial intelligence: DECIDE-AI. Nat. Med..

[19] Hernandez-Boussard T., Bozkurt S., Ioannidis J.P.A., Shah N.H. (2020). MINIMAR (MINimum Information for Medical AI Reporting): Developing reporting standards for artificial intelligence in health care. J. Am. Med. Inform. Assoc..

[20] Klement W., El Emam K. (2023). Consolidated Reporting Guidelines for Prognostic and Diagnostic Machine Learning Modeling Studies: Development and validation. J. Med. Internet Res..

[21] American Medical Association AI Usage Among Doctors Doubles as Confidence in Technology Grows. AMA. https://www.ama-assn.org/press-center/ama-press-releases/ama-ai-usage-among-doctors-doubles-confidence-technology-grows.

[22] Luo X., Wang B., Shi Q., Wang Z., Lai H., Liu H., Qin Y., Chen F., Song X., Ge L. (2025). Lack of methodological rigor and limited coverage of generative artificial intelligence in existing artificial intelligence reporting guidelines: A scoping review. J. Clin. Epidemiol..

[23] Bedi S., Liu Y., Orr-Ewing L., Dash D., Koyejo S., Callahan A., Fries J.A., Wornow M., Swaminathan A., Lehmann L.S. (2025). Testing and evaluation of health care applications of large language models: A systematic review. JAMA.

[24] Aldea M., Salto-Tellez M., Marra A., Umeton R., Stenzinger A., Koopman M., Prelaj A., Kehl K., Gilbert S., Leßmann M.-E. (2025). ESMO basic requirements for AI-based biomarkers in oncology (EBAI). Ann. Oncol..

[25] US Food and Drug Administration (2026). Clinical Decision Support Software: Guidance for Industry and Food and Drug Administration Staff.

[26] US Food and Drug Administration (2025). Good Machine Learning Practice for Medical Device Development: Guiding Principles.

[27] Lekadir K., Frangi A.F., Porras A.R., Glocker B., Cintas C., Langlotz C.P., Weicken E., Asselbergs F.W., Prior F., Collins G.S. (2025). FUTURE-AI: International consensus guideline for trustworthy and deployable artificial intelligence in healthcare. BMJ.

[28] Collins G.S., Dhiman P., Andaur Navarro C.L., Ma J., Hooft L., Reitsma J.B., Logullo P., Beam A.L., Peng L., Van Calster B. (2021). Protocol for development of a reporting guideline (TRIPOD-AI) and risk of bias tool (PROBAST-AI) for diagnostic and prognostic prediction model studies based on artificial intelligence. BMJ Open.

[29] Gallifant J., Afshar M., Ameen S., Aphinyanaphongs Y., Chen S., Cacciamani G., Demner-Fushman D., Dligach D., Daneshjou R., Fernandes C. (2025). The TRIPOD-LLM reporting guideline for studies using large language models. Nat. Med..

[30] CHART Collaborative (2025). Reporting guideline for chatbot health advice studies: The Chatbot Assessment Reporting Tool (CHART) statement. Br. J. Surg..

[31] Park S.H., Suh C.H., Lee J.H., Tejani A.S., You S.C., Kahn C.E., Moy L. (2025). Minimum Reporting Items for Clear Evaluation of Accuracy Reports of Large Language Models in Healthcare (MI-CLEAR-LLM): 2025 Updates. Korean J. Radiol..

[32] Wong E.Y.T., Verlingue L., Aldea M., Franzoi M., Umeton R., Halabi S., Harbeck N., Indini A., Prelaj A., Romano E. (2025). ESMO guidance on the use of large language models in clinical practice (ELCAP). Ann. Oncol..

[33] Guni A., Sounderajah V., Whiting P., Bossuyt P.M., Darzi A., Ashrafian H. (2024). Revised tool for the quality assessment of diagnostic accuracy studies using AI (QUADAS-AI): Protocol for a qualitative study. JMIR Res. Protoc..

[34] Hua P., Olofson A., Farhadi F., Hondelink L., Tsongalis G., Dragnev K., Hoegemann Savellano D., Suriawinata A., Tafe L., Hassanpour S. (2025). Predicting targeted therapy resistance in non-small cell lung cancer using multimodal machine learning. J. Thorac. Dis..

[35] Macheka S., Ng P.Y., Ginsburg O., Hope A., Sullivan R., Aggarwal A. (2024). Prospective evaluation of artificial intelligence (AI) applications for use in cancer pathways following diagnosis: A systematic review. BMJ Oncol..

[36] Causio F., De Vita V., Nappi A., Sawaya M., Rocco B., Foschi N., Maioriello G., Russo P. (2026). Survival prediction in patients with bladder cancer undergoing radical cystectomy using a machine learning algorithm: Retrospective single-center study. JMIR Perioper. Med..

[37] Lee A.S., Valero C., Yoo S.K., Vos J.L., Chowell D., Morris L.G.T. (2024). Validation of a machine learning model to predict immunotherapy response in head and neck squamous cell carcinoma. Cancers.

[38] Huo B., Boyle A., Marfo N., Tangamornsuksan W., Steen J.P., McKechnie T., Lee Y., Mayol J., Antoniou S.A., Thirunavukarasu A.J. (2025). Large language models for chatbot health advice studies: A systematic review. JAMA Netw. Open.

[39] Suh P.S., Jeong S.Y., Ueda D., Shim W.H., Heo H., Woo C.-Y., Park H., Suh C.H. (2026). Insufficient reporting quality in large language model studies in the field of radiology. Insights Imaging.

[40] Ko J.S., Heo H., Suh C.H., Yi J., Shim W.H. (2025). Adherence of studies on large language models for medical applications published in leading medical journals according to the MI-CLEAR-LLM checklist. Korean J. Radiol..

[41] Ali M., Taha Z., Morsey M.M. (2026). Ontology-grounded knowledge graphs for mitigating hallucinations in large language models for clinical question answering. J. Biomed. Inform..

[42] Chen L., Zaharia M., Zou J. (2024). How is ChatGPT’s behavior changing over time?. Harv. Data Sci. Rev..

[43] Lu J.H., Callahan A., Patel B.S., Morse K.E., Dash D., Pfeffer M.A., Shah N.H. (2022). Assessment of adherence to reporting guidelines by commonly used clinical prediction models from a single vendor: A systematic review. JAMA Netw. Open.

